# Atomic-resolution crystal structures of the immune protein conglutinin from cow reveal specific interactions of its binding site with *N*-acetylglucosamine

**DOI:** 10.1074/jbc.RA119.010271

**Published:** 2019-09-27

**Authors:** Janet M. Paterson, Amy J. Shaw, Ian Burns, Alister W. Dodds, Alpana Prasad, Ken B. Reid, Trevor J. Greenhough, Annette K. Shrive

**Affiliations:** ‡ School of Life Sciences, Keele University, Staffordshire ST5 5BG, United Kingdom; § MRC Immunochemistry Unit, Department of Biochemistry, University of Oxford, South Parks Road, Oxford OX1 3QU, United Kingdom

**Keywords:** collectin, complement, crystal structure, carbohydrate-binding protein, host-pathogen interaction, innate immunity, structural biology, lectin, conglutinin, surfactant protein

## Abstract

Bovine conglutinin is an immune protein that is involved in host resistance to microbes and parasites and interacts with complement component iC3b, agglutinates erythrocytes, and neutralizes influenza A virus. Here, we determined the high-resolution (0.97–1.46 Å) crystal structures with and without bound ligand of a recombinant fragment of conglutinin's C-terminal carbohydrate-recognition domain (CRD). The structures disclosed that the high-affinity ligand *N*-acetyl-d-glucosamine (GlcNAc) binds in the collectin CRD calcium site by interacting with the O3′ and O4′ hydroxyls alongside additional specific interactions of the *N*-acetyl group oxygen and nitrogen with Lys-343 and Asp-320, respectively. These residues, unique to conglutinin and differing both in sequence and in location from those in other collectins, result in specific, high-affinity binding for GlcNAc. The binding pocket flanking residue Val-339, unlike the equivalent Arg-343 in the homologous human surfactant protein D, is sufficiently small to allow conglutinin Lys-343 access to the bound ligand, whereas Asp-320 lies in an extended loop proximal to the ligand-binding site and bounded at both ends by conserved residues that coordinate to both calcium and ligand. This loop becomes ordered on ligand binding. The electron density revealed both α and β anomers of GlcNAc, consistent with the added α/βGlcNAc mixture. Crystals soaked with α1–2 mannobiose, a putative component of iC3b, reported to bind to conglutinin, failed to reveal bound ligand, suggesting a requirement for presentation of mannobiose as part of an extended physiological ligand. These results reveal a highly specific GlcNAc-binding pocket in conglutinin and a novel collectin mode of carbohydrate recognition.

## Introduction

The collectin family of mammalian innate immune system proteins recognizes pathogens by the repeating pattern of carbohydrates on their surfaces and organizes their disposal through neutralization, agglutination, and opsonization while recruiting immune system cells for their destruction (1, 2). Collectins are also involved in clearance of apoptotic cells and modulation of inflammatory and immune responses (3, 4). The surfactant proteins SP-A and SP-D are found in the lung where they form the first line of defense against inhaled pathogens and allergens. Mannose-binding lectin (MBL)^5^ is a serum protein which, in common with CL-L1, CL-K1, and CL-P1, is able to activate the complement system to dispose of pathogens (5, 6). In addition to the collectins found in other mammals, the family *Bovidae* has three serum collectins: bovine conglutinin, CL-43, and CL-46. Phylogenetic analysis indicates that these bovine collectins evolved from a common ancestral SP-D gene after the divergence of the *Bovidae* from the mammalian line (7).

The rapid evolution of additional collectins suggests that their presence confers a survival advantage to the animals. The digestive system in ruminants relies on a symbiotic relationship with vast numbers of microorganisms in the rumen, which are able to break down the cellulose in food. The bovine serum collectins provide extra defense against microorganisms which leak into the bloodstream. As well as binding, neutralizing, and opsonizing the microorganisms, it is thought that they lead to reduction in inflammatory responses (7), whereas low conglutinin levels in plasma are associated with increased susceptibility to infection (8). Mice injected with conglutinin followed by *Salmonella typhimurium* showed significantly reduced blood bacterial levels and greater survival compared with controls (9). *In vitro* experiments also demonstrated antibacterial activity of conglutinin against *S. typhimurium* and *Escherichia coli* (9). In addition to this antimicrobial role, conglutinin levels in the *Bovidae* vary with diet, season, infections, and stage of the reproductive cycle (10), with a significant presence in fetal serum (11). The significance of these variations remains unclear.

Bovine serum conglutinin has been shown to bind, through the globular carbohydrate recognition domain (CRD), to specific carbohydrate structures on complement effector protein iC3b, which arises following activation and cleavage of complement component C3 (12). This is thought to enhance complement-dependent clearance through agglutination of complement-reacted microorganisms. The iC3b α-oligosaccharide is of high-mannose type, being (Man)_9_(GlcNAc)_2_-Asn or (Man)_8_(GlcNAc)_2_-Asn (13, 14), and the binding is proposed to be via the terminal α1–2 mannobiosyl unit (15). It is suggested that C3b undergoes a conformational change that exposes its glycan when cleaved (iC3b) leading to conglutinin recognition of iC3b (13). The binding of conglutinin to iC3b is inhibited by *N*-acetylglucosamine (GlcNAc) (12) which, unique to the bovine collectins conglutinin and CL-46, is reported to be the highest affinity ligand, in the case of conglutinin by an order of magnitude relative to other monosaccharide ligands such as *N*-acetyl mannosamine (ManNAc), mannose and glucose and to the maltose disaccharide (16–20). Conglutinin has been shown to bind to some complex-type as well as high mannose-type oligosaccharide chains (21). This is thought to be via terminal GlcNAc or mannose residues, and for the high mannose-type there seems to be a preference for the terminal manα1–2 sequence although there is increased reactivity from the Man_5_ to the Man_8_ structures. Complement-independent anti-mycobacterial activity has also been demonstrated for a recombinant neck plus CRD fragment of conglutinin which is thought to bind directly to *Mycobacterium bovis* Bacille Calmett-Guérin (BCG) lipoarabinomannan in a calcium-dependent manner, inhibiting uptake by macrophages (22). Binding to yeast carbohydrate structures including zymosan and mannan has also been reported (23).

Bovine serum conglutinin has been shown to inhibit influenza A virus (IAV) more efficiently than other collectins, and a functionally intact recombinant homotrimer (neck plus CRDs) (24) has been shown to be active against IAV (25). An engineered SP-D/conglutinin (neck/CRD) fusion protein (26) appears to combine the beneficial properties of the two components with enhanced IAV neutralizing activity, whereas targeted mutation (SP-D R343V) enhances SP-D recognition of high mannose viral glycans (27). Reported ligand-bound crystal structures include CRD and trimeric fragments of MBP/MBL, and trimeric fragments of human SP-D, porcine SP-D, rat SP-A, and CL-K1 (27–37). These structures reveal a conserved calcium-binding site within the CRD as part of the significant sequence conservation throughout the ligand-binding domain of the CRD.

We present here high-resolution crystal structures with and without bound ligand of a recombinant fragment of the C-terminal carbohydrate recognition domain of conglutinin, the first crystal structures of a bovine collectin. The results reveal a highly specific binding pocket for GlcNAc, as opposed to other monosaccharide ligands, and a novel collectin mode of carbohydrate recognition which utilizes interactions which extend beyond the conserved collectin calcium-binding pocket and the binding-site flanking residues.

## Results

### Protein expression and purification

**Figure 1.**
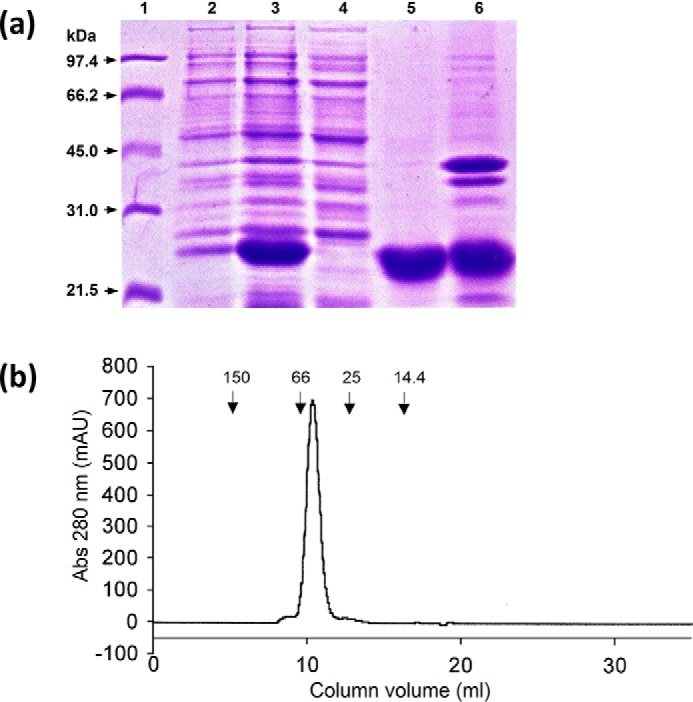
**Expression and purification of rfBC.** *a*, reducing 15% SDS-PAGE analysis of the expression of rfBC. *Lane 1*, low molecular weight markers; *lane 2*, total cell lysate of uninduced cells; *lane 3*, total cell lysate of induced cells harvested 2 h post induction and chilled for 30 min on ice with shaking; *lane 4*, sonicated supernatant; *lane 5*, refolded protein after overnight dialysis; *lane 6*, dialysate pellet containing insoluble material. *b*, gel-filtration chromatography elution profile of rfBC. Following ion exchange purification of the expressed rfBC protein (Q-Sepharose followed by Mono Q) the purified protein was run at a flow rate of 1 ml/min (tris/EDTA buffer) through a Superdex75 gel-filtration column. 0.5 ml volume fractions were collected. The molecular mass standards alcohol dehydrogenase (150 kDa), BSA (66 kDa), carbonic anhydrase (29 kDa), and cytochrome C (14.4 kDa) used for calibration are shown.

**Figure 2.**
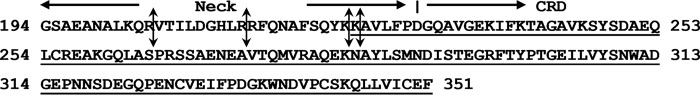
**Sequence of the rfBC recombinant fragment.** The first five amino acids GSAEA (GLAEV in conglutinin) are vector derived. The truncated monomeric fragment used for structure determination is *underlined* with the sites of proteolysis within the α helical coiled coil neck region, leading to the truncated fragment, indicated by the *vertical arrows*.

Expression of the trimeric recombinant neck plus CRD conglutinin fragment provided ∼40 mg protein per liter culture, with almost all the protein in the inclusion bodies as evidenced by the absence of corresponding protein in the sonicate supernatant. The recombinant fragment showed a relative molecular mass of ∼23 kDa under reducing conditions on a 15% (w/v) acrylamide gel, corresponding to the monomeric recombinant neck plus CRD (rfBC) (Fig. 1*a*). The recombinant fragment sequence is shown in Fig. 2.

The protein eluted as a single peak from a Q-Sepharose column and the subsequent Mono Q column at salt concentrations of ∼200 mm and ∼225 mm, respectively. Following ion exchange purification, the MW of rfBC under nondissociating conditions was determined by gel filtration, the recombinant protein eluting as a single peak corresponding to 64 KDa, which indicated the trimeric nature of the recombinant rfBC (Fig. 1*b*). The N-terminal sequence obtained following chromatographic purification and characterization was GSAEANALKQRVTILDGHLRRFQNAFSQYKKAVLFPDGQA, where the first five residues GSAEA are from the vector. Only a single sequence was observed, with the conglutinin sequence starting at Asn-199 in agreement with the published sequence in the protein database (Fig. 2). Cysteine residues are present only in the CRD of the recombinant fragment (Fig. 2).

Crystallization of the purified protein resulted in unliganded and ligand-bound crystal structures of the recombinant fragment of bovine conglutinin which revealed a monomeric CRD rather than the expressed trimeric rfBC. The protein used for crystallization, several months after the initial preparation, was recharacterized by N-terminal sequencing and SDS-PAGE (data not shown). The results showed that 80% of the preparation had the N-terminal sequence starting at conglutinin Lys-224 or Ala-225 whereas the remaining 20% was composed almost equally of material starting at conglutinin Arg-214 and Val-205 (Fig. 2). The SDS gel of the protein also showed that the molecular weight of the majority of the recombinant fragment was less than that expected for the recombinant neck plus CRD monomer. This indicates that the majority of the protein used in the crystallization had lost a substantial amount of the neck region between initial preparation and crystallization and was a monomer of 21.3 kDa, which is consistent with the monomeric CRD fragment seen in the crystal structures.

### Crystal structure

**Figure 3.**
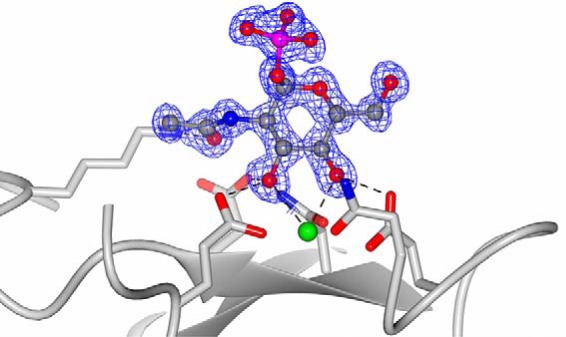
**Electron density for the αGlcNAc-1-P ligand bound to conglutinin.** Conglutinin ligand-binding site with αGlcNAc-1-P ligand shown as *ball-and-stick* model. The 1.0 Å resolution electron density map (*blue*) is 2m*F_o_* − D*F_c_* contoured at 1σ. The calcium ion is in *green* with calcium and ligand coordinating residues Glu-315, Asn-317, Glu-325, and Asn-337 are shown as *stick* models.

Both unliganded and ligand-soaked structures have been determined. The initial structure of the conglutinin CRD showed electron density at the CRD ligand-binding site (Ca1) into which the cryoprotectant, ethylene glycol, was subsequently fitted. A further structure was therefore determined, to a higher resolution of 0.97 Å, utilizing 2,4 methanepentanediol (MPD) as an alternative cryoprotectant. This structure did not show any extra electron density in the ligand-binding site, except for water molecules, and is therefore designated as the native structure. To investigate the structural detail of the known binding properties and specificities of conglutinin (16–20), various ligands which should be able to access the binding site in the crystal were introduced into crystals by adding ligand to cryobuffers. These included GlcNAc, maltoheptaose, inositol 1-monophosphate, α-1,2-mannobiose, and ManNAc. Only the highest affinity ligand, GlcNAc, was visualized in the ligand-binding site along with the closely similar *N*-acetyl-α-d-glucosamine 1-phosphate (αGlcNAc-1-P) (Fig. 3). Initial experiments for α-1,2-mannobiose, implicated in the binding of conglutinin to iC3b, used a ligand molar excess of three times that was successfully used for GlcNAc but did not show any ligand in the crystal. Extending the soaking time and final ligand concentration in the cryobuffers still did not show the ligand in the binding site, or at any other location in the protein. The GlcNAc and αGlcNAc-1-P ligand-bound structures were subsequently refined along with the unliganded and ethylene glycol–bound structures (see Table 1).

**Table 1:** **Crystallographic data and refinement statistics for conglutinin** Figures in parentheses refer to the highest resolution bin. r.m.s.d., root mean square deviation.

	Native conglutinin	Ethylene glycol–bound conglutinin	GlcNAc-bound conglutinin	αGlcNAc-1-P–bound conglutinin
**Data collection**				
Synchrotron station	DLS I03	SRS 14.2	SRS 14.1	DLS I04
Wavelength (Å)	0.920	0.985	1.488	0.827
Temperature (K)	100	100	100	100
Space group	P4_3_	P4_3_	P4_3_	P4_3_
Cell dimensions (Å)	a = 50.17 c = 52.25	a = 50.82 c = 52.84	a = 50.22 c = 51.93	a = 50.28 c = 52.19
Resolution range (Å)	50.0–0.97 (1.02–0.97)	52.9–1.25 (1.29–1.25)	25.1–1.46 (1.54–1.46)	50.3–1.0 (1.004–1.0)
Observations	471,902 (4289)	348,475 (10,133)	116,659 (1258)	538,352 (7243)
Unique reflections	61,261 (1888)	35,164 (2367)	18,301 (714)	70,046 (985)
Completeness (%)	81.2 (20.5)	94.3 (66.9)	80.8 (21.8)	99.7 (99.9)
*R*_merge_*^a^*	0.050 (0.216)	0.034 (0.199)	0.036 (0.121)	0.040 (0.389)
I/σ(I)	7.0 (3.2)	12.3 (3.5)	9.9 (2.8)	9.5 (2.0)
**Refinement**				
Protein atoms	902	946	943	911
Residues chain A	230–317, 324–351	230–351	230–351	230–317, 323–351
Other atoms				
Calcium ions	1	2	2	2
Ligand		4	15 (×2)	19
Water	141	101	149	173
*R*_work_*^b^* (%)	0.135	0.181	0.161	0.124
*R*_free_*^c^* (%)	0.139	0.203	0.184	0.140
r.m.s.d. bond length (Å)	0.01	0.01	0.010	0.011
r.m.s.d. bond angle (°)	1.65	1.68	1.38	1.78
Average B-values (Å^2^)				
Protein	14.60	16.23	14.05	12.99
Water	25.54	24.14	25.05	26.67
Other hetero-atoms	16.95	21.67	15.83	17.01
**Ramachandran plot values (%)***^d^*				
Favored	99.1	96.7	97.5	99.1
Outliers	0.0	0.0	0.0	0.0
**PDB ID**	**6RYG**	**6RYJ**	**6RYM**	**6RYN**

*^a^ R*_merge_ = Σ*_h_*Σ*_j_* |*I_h,j_* − *I_h_*|/Σ*_h_*Σ*_j_* |*I_h,j_*|, where *I_h,j_* is the *j*th observation of reflection *h* and *I_h_* is the mean of the *j* measurements of reflection *h*.

*^b^ R*_conv_ = Σ*_h_* ‖*F_oh_*| − |*F_ch_*‖/Σ*_h_* |*F_oh_*|, where *F_oh_* and *F_ch_* are the observed and calculated structure factor amplitudes, respectively, for the reflection *h*.

*^c^ R*_free_ is equivalent to *R*_conv_ for a 5% subset of reflections not used in the refinement.

*^d^* Defined according to MolProbity.

**Figure 4.**
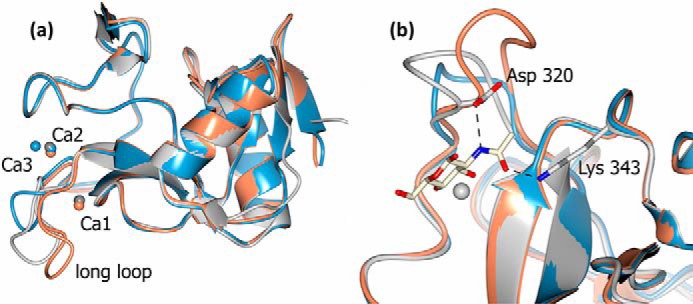
**Structure of the CRD.** Overlay of conglutinin (*gray*), hSP-D (*blue*), and pSP-D (*orange*) CRDs generated by superposing (least squares fit of main chain) conglutinin residues 232–319 and 325–351 with equivalent residues in PDB ID 1PWB and PDB ID 4DN8. *a*, overlay illustrating the location of the calcium ions, Ca1, Ca2, and Ca3, with Ca3 only found in hSP-D. The *long loop* indicated corresponds to residues 318–324 in conglutinin. *b*, GlcNAc-bound conglutinin structure with ligand coordinating residues Asp-320, from the long loop, and Lys-343 shown as *sticks*. For clarity only βGlcNAc and conglutinin Ca1 are shown.

**Figure 5.**
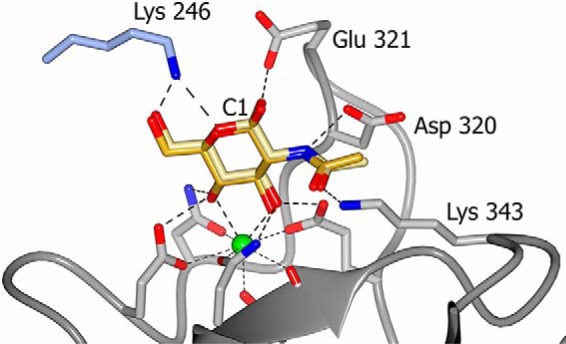
**GlcNAc binding in conglutinin CRD ligand site showing key amino acids and interactions between bound ligand and protein.** Both αGlcNAc and βGlcNAc are seen in the binding site with the anomeric carbon C1 indicated. Amino acid interactions are shown for αGlcNAc. βGlcNAc interactions are the same except for that with Glu-321 which is only present for αGlcNAc. The Lys-246 interacting residue from a neighboring subunit via a crystal contact is shown in *blue* and calcium ion Ca1 is shown as a *green sphere*.

The conglutinin CRD monomer has an overall topology similar to that of the known collectin structures (Fig. 4). In all structures, calcium Ca1 is present at the collectin CRD ligand-binding site. Additionally, except for the structure without bound ligand, there is evidence of a calcium ion in the collectin Ca2 site although the electron density and the average B-factors (Ca1 14.0 Å^2^, Ca2 33.0 Å^2^) suggest this site is not as fully occupied as Ca1, particularly for the ethylene glycol and αGlcNAc-1-P structures. In the unliganded structure, the lack of electron density for the calcium ion at the Ca2 site is accompanied by a lack of electron density, or movement away from the site, for some of the side chains of the Ca2 coordinators. There is no electron density in any of the structures to support the presence of the third calcium ion observed in some collectins. Ca1 is coordinated in a similar way to other structurally characterized collectins (Fig. 5 and Table 2). Protein coordination to Ca1 is by Glu-315, Asn-317, Glu-325, Asn-337, Asp-338, and the carbonyl O of Asp-338. In the ligand-bound structures Ca2 is coordinated by Asp-291 (2), Glu-295 (2), Asn-318 (this interaction is not present in the αGlcNAc-1-P–bound structure), Asn-326, the carbonyl O of Glu-325, and one water molecule.

**Table 2:** **Calcium- and ligand-binding distances (Å)** Lys-246 refers to a residue from a symmetry related molecule in the crystal. W corresponds to water molecule. Dash means no interaction.

Atom 1	Atom 2	Native conglutinin	Ethylene glycol ligand	αGlcNAc ligand	βGlcNAc ligand	αGlcNAc-1-P ligand
Ca1	Glu-315 OE1	2.49	2.62	2.62	2.62	2.56
	Asn-317 OD1	2.44	2.48	2.44	2.44	2.43
	Glu-325 OE1	2.43	2.53	2.39	2.39	2.44
	Asn-337 OD1	2.42	2.51	2.43	2.43	2.41
	Asp-338 OD1	2.27	2.34	2.30	2.30	2.32
	Asp-338 O	2.56	2.61	2.48	2.48	2.51
	Ligand					
	O_A_	—	O1′: 2.57	O3′: 2.76	O3′: 2.41	O3′: 2.58
	O_B_	—	O2′: 2.52	O4′: 2.52	O4′: 2.37	O4′: 2.50
	W					
		2.46	—	—	—	—
		3.50	—	—	—	—
Ligand						
O_A_	Glu-325 OE2	—	O1′: 2.60	O3′: 2.42	O3′: 2.54	O3′: 2.60
	Asn-337 ND2	—	O1′: 3.05	O3′: 2.92	O3′: 2.84	O3′: 2.92
O_B_	Glu-315 OE2	—	O2′: 2.69	O4′: 2.70	O4′: 2.59	O4′: 2.62
	Asn-317 ND2	—	O2′: 3.00	O4′: 3.07	O4′: 2.90	O4′: 3.29
O7′	Lys-343 NZ	—	—	2.79	2.83	2.87
N	Asp-320 OD1	—	—	3.04	3.18	—
O1′	Glu-321 OE1	—	—	2.88	4.87	—
O5	Lys-246 NZ	—	—	2.97	3.09	3.15
O5	W	—	—	3.14	3.11	3.04
O6′	Lys-246 NZ	—	—	2.89	2.72	2.92
O6′	W	—	—	2.51	3.03	3.05 & 2.75
OP1	Lys-246 NZ	—	—	—	—	2.85
Ca2	Asp-291 OD1	—	2.86	2.66	2.66	2.81
	Asp-291 OD2	—	2.33	2.33	2.33	2.26
	Glu-295 OE1	—	3.12	2.57	2.57	2.82
	Glu-295 OE2	—	2.72	2.56	2.56	2.81
	Asn-318 OD1	—	3.14	2.65	2.65	—
	Glu-325 O	—	2.83	2.64	2.64	2.91
	Asn-326 OD1	—	3.04	2.79	2.79	3.15
	W	—	2.30	2.33	2.33	2.09
Glu-329						
OE1	Lys-343 NZ	2.77	2.83	2.79	2.79	2.76
OE2	Asn-337 ND2	2.82	2.91	2.85	2.85	2.80

### Ligand-binding site

**Figure 6.**
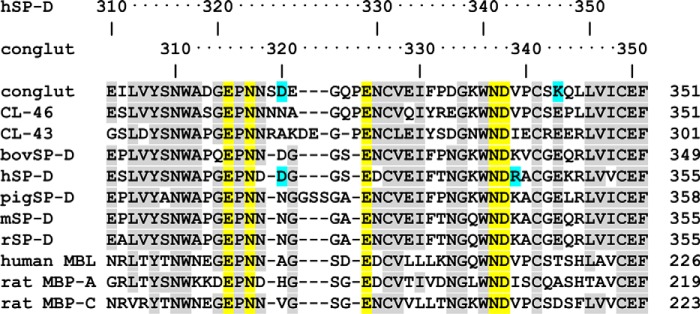
**Sequence alignment in the calcium- and ligand-binding region in the collectin CRD.** Conserved residues are shaded *gray* and conserved Ca1 coordinators are *yellow*. The specific GlcNAc conglutinin *N*-acetyl interacting residues Asp-320 and Lys-343 along with the binding site flanking residues of hSP-D, Asp-325, and Arg-343 (see Shrive *et al.*, 32) are highlighted in *cyan*.

Adjacent to the calcium CRD site, the longer of the two loops involved in binding to the calcium ions (see Fig. 4) has an insertion compared with the homologous collectin human surfactant protein D (hSP-D) (Fig. 6). These additional two residues compared with hSP-D result in an extended loop near the CRD ligand–binding site, in a similar manner to pig surfactant protein D (pSP-D). In the unliganded and αGlcNAc-1-P–bound structures this Asn-318–Pro-324 loop is disordered (318–323). Upon binding of the ligand GlcNAc or ethylene glycol the 318–324 loop becomes ordered, although the electron density is not as clearly defined as in the majority of the CRD structure. Within the structure without bound ligand there is some ill-defined electron density for the loop that could correspond to the ligand-bound loop, suggesting a small degree of order. However, it would not have been possible to discern the location of the loop without the prior knowledge of the position of the loop in the ligand-bound structures, supporting that the loop is mobile in the absence of ligand.

**Figure 7.**
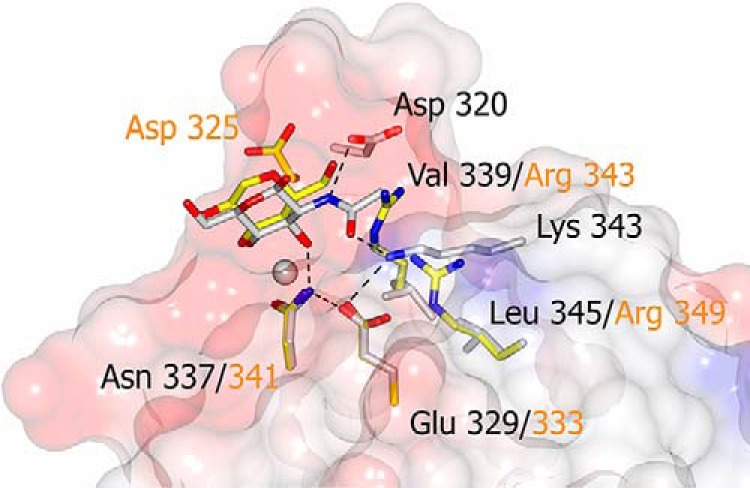
**Extended binding site interactions.** Conglutinin CRD (surface view) with residues in the extended ligand-binding pocket highlighted in *gray* with *black labels*. Corresponding residues in hSP-D (least squares fit of 1PWB CRD to main chain conglutinin residues 232–319 and 325–351) are shown in *yellow* with *orange labels*.

The GlcNAc-bound structure shows evidence of both α and β anomers present in the electron density (Fig. 5), reflecting the heterogeneous mixture of the commercially sourced GlcNAc. GlcNAc interacts with the CRD calcium and its coordinators Glu-315 OE2, Asn-317 ND2, Glu-325 OE2, and Asn-337 ND2 via the O3′ and O4′ hydroxyls, with Asp-320 from the extended loop interacting with the N group of GlcNAc (α 3.04 Å; β 3.18 Å) (Table 2). Additionally, Lys-343 NZ interacts with O7 on the acetyl head group (2.79–2.83 Å) (Fig. 5). Lys-343 adopts the same conformation in all the structures, being held in place by Glu-329, which in turn is interacting with Asn-337 which coordinates Ca1 and hydroxyl of bound ligand (Fig. 7). There is some evidence in the electron density of an interaction between Glu-321 in the extended loop and O1′ of the α anomer of GlcNAc. In addition to the CRD interactions with the *N*-acetyl group, Lys-246 from a symmetry-related molecule interacts with GlcNAc O5 and O6′ (Fig. 5). This residue is in a similar orientation in all the structures determined, including that without ligand, regardless of interactions between protein and ligand. The αGlcNAc-1-P–bound structure shows ligand binding in the calcium site and the additional protein-ligand interaction with Lys-343 as seen for GlcNAc. Residues 318–323 in the extended loop are disordered in the αGlcNAc-1-P structure, the bulky α-linked phosphate precluding the interaction of Asp-320 with the ligand nitrogen (Fig. 3) and preventing the loop adopting the orientation seen in the other ligand-bound structures.

The structure of the conglutinin CRD with ethylene glycol from the cryoprotectant bound at the ligand-binding site reveals ligand binding in a similar manner to that seen for all collectin ligands to date. Two hydroxyls from ethylene glycol, which have the same configuration as the O3′ and O4′ mannose-type pair, interact with the binding-pocket residues and Ca1. For this small ligand no further interactions with protein are seen. Although the 318–324 loop in the ethylene glycol–bound structure makes no direct interactions with the ligand, the loop is ordered and adopts the same overall conformation as that seen in the GlcNAc-bound structure.

## Discussion

The trimeric recombinant fragment comprises amino acids 199–351 of conglutinin, the N-terminal sequence of the preparation being confirmed by sequencing. The crystal structure however reveals only the monomeric CRD, with electron density definition at the N terminus starting at around amino acid 230 (the residue neighboring proline 229 at the neck-CRD interface, Figs. 2 and 3). Subsequent N-terminal sequencing of the protein used for crystallization revealed that the majority (∼80%) starts at amino acid 224/225 (KAVLFP/AVLFP; Fig. 2) consistent with the crystal structure. The remaining 20% gave an N-terminal residue of either Arg-214 or Val-205 (Fig. 2). A significant portion of the recombinant chain (25/26 residues for the major component) is believed to have been proteolytically cleaved just prior to the neck-CRD interface because of traces of proteases in the preparation. The time delay of several months between preparation and use, for crystallization, may also be a contributory factor. The absence of the trimerizing neck residues, known to be essential for trimer formation (24, 38) results in the dissociation of the three chains of rfBC to give the monomeric CRD observed here.

The conglutinin CRD fold is very similar to the other known collectin structures (Fig. 4). The overall structure is stabilized by the two typical collectin CRD intermolecular disulfide bridges, Cys-255–Cys-349 and Cys-327–Cys-341 here (Fig. 2). No other cysteines are present in the expressed fragment providing no opportunity for intrachain disulfides to be formed either in the full expressed trimeric fragment (Fig. 1*b*) or the cleaved fragment present during crystallization. Calcium Ca1 at the CRD ligand site is coordinated by conserved collectin residues. The second calcium ion Ca2 is not as tightly bound as Ca1 (as evidenced by higher B-factor compared with Ca1) and is not observed in the structure without bound ligand. This may be because of a number of interdependent factors including the loss of order of 318–323 in the calcium binding loop, loss of Ca2 coordination by Asn-318, the lack of an interaction between Asp-320 and ligand, and low calcium concentration in the crystallization buffer. A third calcium ion is not present in any of the conglutinin CRD data consistent with the presence of Asn at the Ca2/Ca3 coordinating residue conglutinin 326 rather than the Asp present in rat MBP-A, hSP-D, and human MBL which all contain a third calcium ion (Fig. 6).

Although the structures of the conglutinin CRD reveal ligand binding to calcium in a similar manner to that seen for all collectin ligands to date, with two hydroxyls which have the same configuration as the O3′ and O4′ hydroxyls of mannose/glucose-type rings interacting with Ca1, there are additional specific interactions with the high-affinity GlcNAc ligand. The *N*-acetyl head group interacts with Lys-343 (via O7′) and with Asp-320 (via N) in the extended conglutinin loop. Conglutinin does not discriminate between the α/β GlcNAc anomers, with the crystal structure suggesting that both are bound with similar occupancy despite an indication of an additional protein-ligand interaction involving Asp-321 and the anomeric hydroxyl O1′ in the α anomer. As this anomer is only partially occupied (as evidenced by the presence of both α and β forms in the structure), this may explain why the Glu-321 density is not as clearly defined as may be expected for an interacting residue.

The head group NZ of conglutinin Lys-343 which interacts with GlcNAc O7′ is held in position by Glu-329 which is linked to the collectin-conserved Asn-337 which coordinates the CRD calcium Ca1 and hydroxyl of bound ligand (Figs. 6 and 7). The Lys-343 occupies a position between the small, hydrophobic residues Val-339 and Leu-345 (Fig. 7) which are replaced in hSP-D by Arg-349 (hSP-D numbering) and the key flanking residue Arg-343. Thus in conglutinin the flanking residue Val-339, equivalent to SP-D Arg-343, indirectly influences, and enhances, GlcNAc binding by allowing O7′ of the acetyl head group direct access to Lys-343 NZ (NZ-O7′ 2.79–2.87 Å) (Fig. 7). Positioning GlcNAc in hSP-D in a similar manner to that observed here would give a clash between the hSP-D Arg-343 head-group and ligand, specifically the acetyl head group of GlcNAc (39) which is presumably why ManNAc is rotated 180° when bound to hSP-D (29). This would also be true for pSP-D which has Lys and Arg in equivalent positions to hSP-D Arg-343 and Arg-349 (Fig. 6).

The specificity of conglutinin for GlcNAc provided by the Lys-343 to *N*-acetyl binding alongside the standard collectin interactions of the Glc O3′ and O4′ hydroxyls with calcium and protein, is enhanced by the extended loop Asn-318–Pro-324, bounded at both ends by conserved residues which coordinate both ligand and Ca1, which becomes ordered on GlcNAc ligand binding, with Asp-320 OD1 making a specific interaction with the *N*-acetyl nitrogen (Fig. 5). The structure of the αGlcNAc-1-P complex again shows interaction with Lys-343 in addition to Ca1 binding-site residues, but with the extended flexible loop displaced by the phosphate group such that Asp-320 is no longer within binding distance. The extended flexible loop in pSP-D (insertion of GSS; Figs. 4 and 6) also becomes more ordered on sugar binding, although no specific interactions between the loop and bound ligand are reported (40, 41), whereas the RAK insertion in CL-43 is reported to enhance specificity for mannose-rich viral glycoconjugates (42).

The combination of a flanking valine (339) with Lys-343 and Asp-320 appears to be a key element in the recognition and binding of GlcNAc by conglutinin with an affinity order of magnitude higher than other monosaccharides (16–20). The only other collectin where GlcNAc is reported to bind with high affinity (7) is CL-46 which also has a flanking valine, however the potential GlcNAc *N*-acetyl coordinating residues, in equivalent positions to conglutinin Lys-343 and Asp-320, are glutamate and asparagine, respectively (Fig. 6). Several other collectins have specificity, albeit usually weak, for GlcNAc ligands (16, 17), the known structures with bound GlcNAc or GlcNAc type terminal ligand revealing the orientation of the bound sugar ring as generally reversed to that observed in the conglutinin structure here. This is the case for two of the three subunits in the GlcNAc–rat MBP-A trimeric complex, for GlcNAc–rat MBP-C (33, 34), and for ManNAc–hSP-D (29). The exception is the C subunit of rat MBP-A (34) which shows the same GlcNAc orientation as conglutinin, although this is thought to be because of crystal contacts and there are no specific interactions between the protein and the acetyl group. Although the ability of crystal contacts to influence ligand orientation is well-documented (32), the interaction here of Lys-246 from a neighboring subunit with GlcNAc O5 and O6′ does not appear to influence ligand orientation, protein-GlcNAc binding determinants (Glu-315, Asn-317, Glu-325, Asn-337, Asp-320, Ca1, Lys-343), or the extended loop 318–324, because each of these for GlcNAc is closely similar to those in both the unliganded and the ethylene glycol–bound structures where the Lys-246–ligand interaction is absent. The ligand-binding site is formed ready to interact with the incoming ligand, the NZ of the contact residue Lys-246 then shifting by ∼2 Å to interact with the bound GlcNAc ligand.

The biological and physiological relevance of the high specificity for the *N*-acetyl group, and for GlcNAc in particular, is far from clear. GlcNAc→Glc represents the conserved motif hexose-1→2-hexose in cores of many *Enterobacteriaceae*, including all *Salmonella,* which share a common major core structure (that of the Ra mutant), and *E. coli* R2 and K12 (43–45). Where the LPS is extended to O antigen the Ra GlcNAc is present as a branch linked through O1′ to the LPS main chain glucose and is thus available for recognition by conglutinin. The ability of the conglutinin-binding pocket to accommodate the phosphate in GlcNAc-1-P is an important indicator that 1-linked GlcNAc such as that of the *Salmonella* LPS can be recognized and bound by conglutinin. This is consistent with the *in vitro* and *in vivo* studies which demonstrate the antibacterial activity of conglutinin against *S. typhimurium* and *E. coli* (8, 9).

Conglutinin, which is known to bind to high mannose-type oligosaccharide with a preference for the terminal manα1–2 sequence (21), binds to the C3-derived glycoprotein iC3b present on pathogens and facilitates clearance (13–15), with binding reported to be exclusively mediated through the *N*-linked Man oligosaccharides on the iC3b α chain (23, 46). In addition, binding to yeast mannan and complement-activated immune complexes has been shown to be calcium-dependent and inhibited by GlcNAc (12), suggesting a central role for the CRD-binding site. In our studies, soaking of α1–2-mannobiose into the unliganded crystals, successfully demonstrated for hSP-D (32), failed to reveal any ligand in the CRD site (or elsewhere) even at high concentrations. Because the binding site appears to be openly accessible in the crystal, the failure to bind this simple mannobiose suggests a requirement for the iC3b α chain oligosaccharide to be presented by the carrier protein iC3b in a conformation which enables high-specificity binding (46, 23), perhaps utilizing an extended binding site as for *Salmonella enterica* Minnesota R5 LPS (36). This is supported by Mizuochi *et al.* (21), who report that high mannose-type oligosaccharides exhibit increased reactivity from the Man_5_ to the Man_8_ structures. Calcium-dependent binding of *M. bovis* Bacille Calmett-Guérin by conglutinin is also thought to be via terminal dimannose units of the bacterial lipoarabinomannan (22), our inability to visualize binding of simple α1–2-mannobiose in the openly accessible ligand-binding site further suggesting a requirement for presentation of dimannose as part of extended physiological ligands. The primary biological roles of bovine conglutinin remain to be determined as do the details of the binding interactions with iC3b, bacterial LPS, and viral glycans. The uniquely designed, highly specific GlcNAc-binding pocket and the ability of GlcNAc to abolish binding to iC3b suggest a dual role for conglutinin, directly binding to iC3b via the high mannose oligosaccharide attached to the N-terminal of the α chain and directly binding pathogens via exposed and accessible GlcNAc on the pathogen surface. The competition between these two conglutinin targets for the Ca1-binding site suggests overlap between the two recognition sites. Whether conglutinin can simultaneously bind, via different homotrimeric CRDs, to both iC3b coated microorganisms and to the microorganism itself is not at all clear, but it would be surprising if the exquisitely designed GlcNAc-binding site was not central to the associated biological roles.

## Experimental procedures

### Protein expression

Expression of the recombinant fragment (rf) of bovine conglutinin (BC) consisting of the CRD and neck regions (rfBC, residues 199–351 of native conglutinin) followed the procedure described by Prasad (47) which is based on the initial cDNA clone of Lu *et al.* (48) and the transformation into competent *E. coli* DH5α cells as described by Wang *et al.* (24). Briefly, the clone was purified from DH5α and used to transform competent NovaBlue cells for maintaining the plasmid. A single colony was picked from a LB agar plate (containing 50 μg/ml carbenicillin and 34 μg/ml chloramphenicol) streaked with BL21(DE3)pLysS containing the recombinant plasmid and was used to inoculate 10 ml of LB media. The culture was incubated overnight at 37 °C with vigorous shaking (225 rpm). Cells were harvested next day at 2000 × *g* and washed once in fresh LB media. The cell pellet was then resuspended into 5 ml of fresh media and used as inoculation for 1 liter of fresh LB media containing the antibiotics chloramphenicol (34 μg/ml) and carbenicillin (50 μg/ml) and incubated at 37 °C with vigorous (225 rpm) shaking.

The cells were induced at *A*_600_ of 0.6 with isopropyl-β-d-thiogalactopyranoside (IPTG, NOVA Biochem), to a final concentration of 0.5 mm, and incubated further for 2 h. The culture was then chilled on ice for 30 min with gentle shaking. The cells were harvested at 8000 × *g* for 10 min at 4 °C and washed once with fresh media. Samples were analyzed on SDS-PAGE. Samples were heated to 100 °C for 5 min (10 min for total cell lysates). 20 μl of each sample was loaded onto the wells and run on a 15% (w/v) acrylamide gel.

### Protein extraction

The cell pellet was resuspended in 25 ml of ice-cold cell-lysis buffer (50 mm Tris-HCl (pH 8.0), 150 mm NaCl, 5 mm CaCl_2_) and frozen at −80 °C. The cells were thawed on ice at 4 °C and the remaining 25 ml of lysis buffer containing Triton X-100 (final concentration 0.05% v/v), DNase (5 μg/ml), MgCl_2_ (0.04 m), and proteases inhibitor mixture tablet was added. The thawed cell suspension was shaken at 4 °C for 45 min. The lysate was then sonicated with 10 30-s pulses of 16 μs amplitude at 15-s intervals. After sonication, the cell soup was centrifuged at 15,000 × *g*. The protein, pelleted as inclusion bodies, was washed twice with ice-cold deionized water to remove traces of Triton X-100.

The pellet was then subjected to a denaturing and refolding procedure, involving repetitive dialysis against concentrations of urea decreasing from 6 m to zero. Before use, all urea solutions were passed through Duolite MB6113 Indicator Mixed Resin (BDH) to remove traces of cyanate ions, which may irreversibly bind to the protein and affect function. The pellet was resuspended in dialysis buffer (20 mm Tris-HCl, 150 mm NaCl, 5 mm CaCl_2_, and 5% v/v glycerol) containing 6 m urea and shaken for 30 min at 4 °C. The denatured protein solution was centrifuged to remove insoluble material. An activated dialysis membrane tubing (Medicell 10 K MWCO) was filled with the cleared supernatant and placed in 2 liters of dialysis buffer containing 4 m urea. The protein was dialyzed against decreasing concentrations of urea with buffer changes every 2 h followed by an additional 24-h dialysis to remove traces of urea. The dialysate was then centrifuged at 15,000 × *g* to remove contaminating aggregates. Samples were analyzed on SDS-PAGE for purity as described elsewhere.

### Protein purification and characterization

Protein samples were passed through Anotope 0.2 μm filters (Whatman) prior to loading. Purification was performed on a Pharmacia Biotech AKTA purifier FPLC system.

### Ion-exchange purification

20 ml of the dialyzed recombinant protein solution was diluted to 50 ml with water and loaded at 2 ml/min onto a Fast Flow Q-Sepharose (16 × 200 mm) column in 20 mm Tris-HCl buffer containing 5 mm CaCl_2_ and 50 mm NaCl. A linear gradient, 0–50% buffer B (buffer A containing 500 mm NaCl), for 90 min was used to elute the protein in 2-ml volume fractions. The fractions were analyzed by SDS-PAGE on 15% (w/v) and the peak fractions pooled. 10 ml of the sample from the Q-Sepharose, diluted to 50 ml with water to reduce the salt concentration, was loaded onto a 5 × 50 mm Mono Q column at 1 ml/min. A steep linear gradient 0–100% buffer B was applied over 5 min. 1-ml fractions were collected and analyzed by 15% SDS-PAGE.

### Gel-filtration chromatography

The peak fractions from Mono Q were loaded onto a prepacked Superdex-75 (HR 10/300 mm) column, 200-μl protein samples were loaded onto the column in Tris buffer, containing 10 mm EDTA. The flow rate was 1 ml/min and 0.5-ml fractions were collected. The column was calibrated with alcohol dehydrogenase (150 kDa), BSA (66 kDa), carbonic anhydrase (29 kDa), and cytochrome C (14.4 kDa).

### N-terminal sequencing

The protein samples were run on a 4–12% (w/v) bis-tris NuPAGE precast gel (Novex UK Ltd) at 200 mA per gel using a Novex XCell II Mini-cell gel apparatus. The gel was electroblotted to ProBlott membrane (PE Applied Biosystems) in a Bio-Rad Trans-Blot electrophoretic transfer cell. The membrane was stained with Coomassie Brilliant Blue. The rfBC band was excised and sequenced using Edman degradation on an Applied Biosystems 494A Procise protein sequencer (PE Applied Biosystems UK). The samples were run on the sequencer by applying to a glass fiber disc pretreated with polybrene to limit sample washout.

### Crystallization and data collection

Crystals of conglutinin were grown in sitting drops consisting of an equal volume (2 μl) of protein solution (8 mg ml^−1^ protein in 13 mm Tris, 100 mm NaCl, 7 mm CaCl_2_, pH 7.5) and precipitant buffer (2.5 m (NH_4_)_2_SO_4_, 0.1 m Tris, pH 8.0–8.5). For the initial data, crystals were prepared for cryocooling using ethylene glycol in precipitant buffer. Successive addition of 2-μl aliquots of increasing concentrations (5, 10, 15, 20%) of ethylene glycol cryobuffer were added to the well, followed by addition of a further 2-μl aliquot of 20% cryobuffer, and an exchange of 10 μl of the resulting well buffer with 20% cryobuffer.

Further experiments indicated the crystals lasted longer in 2–4 methanepentanediol and this was then used as the cryoprotectant for a further unliganded data set and for all the ligand-bound data, with ligands introduced into crystals by their inclusion in the cryobuffer. Ligand concentrations in the cryobuffers for those ligand soaks that showed bound ligand in electron density maps were 10 mm for GlcNAc and 0.1 m for αGlcNAc-1-P, with CaCl_2_ added to give a final concentration of 10 mm in the cryobuffers. All ligands were purchased from Sigma. Where ligand could not be identified in electron density maps, extended soaking times at higher molarity were followed by further data collection. All data were collected either at Daresbury SRS or Diamond Light Source (DLS). Integrated intensities were calculated with the program MOSFLM (49) and data processed using CCP4 suite (50). Data collection and processing statistics are given in Table 1.

### Structure solution and refinement

The structure of conglutinin without bound ligand was solved by molecular replacement with AMoRe (51) using the homologous hSP-D structure (PDB ID 1PW9) as a search model. The recombinant homotrimeric fragment of conglutinin consisted of the C-terminal 153 residues (Asn-199 to Phe-351). The size of the unit cell indicated that a trimer would not fit into the unit cell and there was space only for a monomeric unit. The neck region of collectin molecules is required for trimerization (24, 38), suggesting that some or all of the expressed neck region (Fig. 2) was absent from the crystallized protein. This was confirmed by subsequent recharacterization of the protein used for crystallization by N-terminal sequencing and SDS-PAGE.

Only the CRD portion (residues hSP-D 236 to 355 which correspond to conglutinin residues 230 to 351) of the native recombinant fragment hSP-D structure (PDB ID 1PW9) was thus used as a molecular replacement model. One clear rotation solution was found in AMoRe and translation functions indicated a space group of P4_3_. Initial electron density maps (50) indicated the CRD portion only was present in the structure. Isomorphism was sufficient to allow the coordinates of the unliganded conglutinin CRD structure to be used as a starting model for the ligand-bound structures. The ligand-soaked structures which gave no sign of ligand in the electron density were not refined further. Cycles of iterative model building and refinement were performed; early work on the ethylene glycol and GlcNAc-bound structures used O (52) and CNS (53), however COOT (54) and *REFMAC5* (55) were subsequently used throughout for refinement of these and all further structures. Final models were checked via MolProbity (56) and using Protein Data Bank validation software. For all the structures, the first few residues at the N terminus are not visible in the electron density, definition starting at amino acid 230 (Fig. 2). Final details of the refinement are given in Table 1. There are no residues in the outlier region of the Ramachandran plot (MolProbity). Figures were generated using CCP4 mg (57).

Coordinates and structure factors have been deposited in the Protein Data Bank with accession codes 6RYG (unliganded), 6RYJ (ethylene glycol bound), 6RYM (GlcNAc bound), and 6RYN (αGlcNAc-1-P bound).

## Author contributions

J. M. P., A. J. S., I. B., A. W. D., A. P., T. J. G., and A. K. S. formal analysis; J. M. P., A. J. S., A. W. D., A. P., T. J. G., and A. K. S. validation; J. M. P., A. J. S., I. B., A. W. D., A. P., K. B. R., T. J. G., and A. K. S. investigation; J. M. P., A. J. S., A. W. D., A. P., T. J. G., and A. K. S. visualization; J. M. P., A. J. S., I. B., A. W. D., A. P., K. B. R., T. J. G., and A. K. S. methodology; J. M. P., A. J. S., A. W. D., and T. J. G. writing-original draft; J. M. P., A. J. S., I. B., A. W. D., A. P., K. B. R., T. J. G., and A. K. S. writing-review and editing; I. B., A. W. D., K. B. R., T. J. G., and A. K. S. resources; I. B., A. W. D., A. P., T. J. G., and A. K. S. data curation; I. B., A. W. D., K. B. R., T. J. G., and A. K. S. supervision; A. P., K. B. R., T. J. G., and A. K. S. conceptualization; K. B. R., T. J. G., and A. K. S. funding acquisition; K. B. R. and A. K. S. project administration; A. K. S. software.

## Supplementary Material

Supporting Information

## Footnotes

5 The abbreviations used are:

MBL: mannose-binding lectin
CRD: carbohydrate recognition domain
ManNAc: *N*-acetyl mannosamine
GlcNAc: *N*-acetyl glucosamine
LPS: lipopolysaccharide
IAV: influenza A virus
rfBC: recombinant CRD and neck region of conglutinin
αGlcNAc-1-P: *N*-acetyl-α-d-glucosamine 1-phosphate
SP: surfactant protein
MBP: mannose binding protein.
