# Supplementary material for: Atomic-resolution crystal structures of the immune protein conglutinin from cow reveal specific interactions of its binding site with N-acetylglucosamine
Source: J Biol Chem. 2019 Sep 27;294(45):17155–65. doi: 10.1074/jbc.RA119.010271 (PMC6851296; doi:10.1074/jbc.RA119.010271)
Supplement: Supporting Information [file supp_294_45_17155__index.html]

Atomic-resolution crystal structures of the immune protein conglutinin from cow reveal specific interactions of its binding site with N-acetylglucosamine — Crystal structures of native and ligand-bound conglutinin — Atomic-resolution crystal structures of the immune protein conglutinin from cow reveal specific interactions of its binding site with N-acetylglucosamine — Crystal structures of native and ligand-bound conglutinin — Supporting Information 

# Atomic-resolution crystal structures of the immune protein conglutinin from cow reveal specific interactions of its binding site with *N*-acetylglucosamine

## Supporting Information

- Supporting Information (to be published online) - Supplementary figure
