# Supplementary material for: Atomic-resolution crystal structures of the immune protein conglutinin from cow reveal specific interactions of its binding site with N-acetylglucosamine
Source: J Biol Chem. 2019 Sep 27;294(45):17155–65. doi: 10.1074/jbc.RA119.010271 (PMC6851296; doi:10.1074/jbc.RA119.010271)
Supplement: Supporting Information [file supp_RA119.010271_154527_1_supp_400861_pydndx.pdf]

Supporting information – Figure S1

Atomic resolution crystal structures of bovine conglutinin reveal specific binding site interactions for N-acetyl-glucosamine

Janet M. Paterson[1$], Amy J. Shaw[1$], Ian Burns[1], Alister W. Dodds[2], Alpana Prasad[2£], Ken B. Reid[2#], Trevor J. Greenhough[1] and Annette K. Shrive[1*]

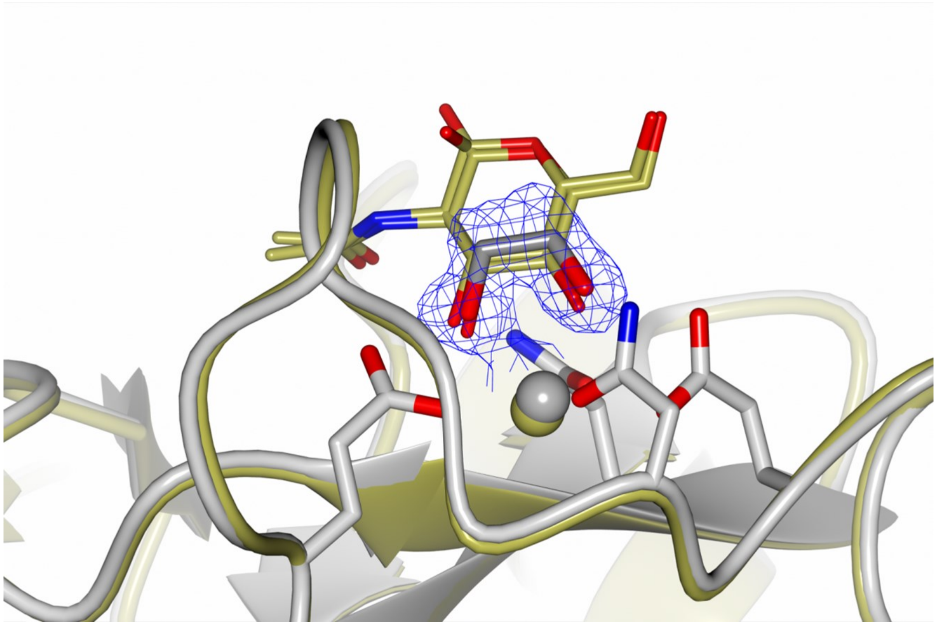

Figure S1. **Ethylene glycol-bound structure.** Structure of ethylene glycol-bound conglutinin (grey) with electron density for the ethylene glycol molecule in the conglutinin binding site shown in blue. The 1.25 Å resolution electron density map is 2mFo-DFc contoured at 1σ. For comparison the GlcNAc-bound conglutinin structure (gold) is overlaid (using secondary structure elements overlay facility in CCP4MG (57)).
